# Lamina-specific contribution of glutamatergic and GABAergic potentials to hippocampal sharp wave-ripple complexes

**DOI:** 10.3389/fncir.2014.00103

**Published:** 2014-08-25

**Authors:** Jan Schönberger, Andreas Draguhn, Martin Both

**Affiliations:** Institute of Physiology and Pathophysiology, University of HeidelbergHeidelberg, Germany

**Keywords:** high-frequency oscillations, memory, synchronization, reactivation, excitation-inhibition interaction

## Abstract

The mammalian hippocampus expresses highly organized patterns of neuronal activity which form a neuronal correlate of spatial memories. These memory-encoding neuronal ensembles form on top of different network oscillations which entrain neurons in a state- and experience-dependent manner. The mechanisms underlying activation, timing and selection of participating neurons are incompletely understood. Here we studied the synaptic mechanisms underlying one prominent network pattern called sharp wave-ripple complexes (SPW-R) which are involved in memory consolidation during sleep. We recorded SPW-R with extracellular electrodes along the different layers of area CA1 in mouse hippocampal slices. Contribution of glutamatergic excitation and GABAergic inhibition, respectively, was probed by local application of receptor antagonists into s. radiatum, pyramidale and oriens. Laminar profiles of field potentials show that GABAergic potentials contribute substantially to sharp waves and superimposed ripple oscillations in s. pyramidale. Inhibitory inputs to s. pyramidale and s. oriens are crucial for action potential timing by ripple oscillations, as revealed by multiunit-recordings in the pyramidal cell layer. Glutamatergic afferents, on the other hand, contribute to sharp waves in s. radiatum where they also evoke a fast oscillation at ~200 Hz. Surprisingly, field ripples in s. radiatum are slightly slower than ripples in s. pyramidale, resulting in a systematic shift between dendritic and somatic oscillations. This complex interplay between dendritic excitation and perisomatic inhibition may be responsible for the precise timing of discharge probability during the time course of SPW-R. Together, our data illustrate a complementary role of spatially confined excitatory and inhibitory transmission during highly ordered network patterns in the hippocampus.

## Introduction

The hippocampus expresses a variety of highly ordered spatiotemporal activity patterns which are believed to underlie memory formation and memory consolidation (Buzsáki, 1989; Harris et al., 2003; Buzsáki and Draguhn, 2004). During immobility and slow-wave sleep of rodents, the CA3 network generates repetitive bursts of activity which propagate along CA1 and the subiculum towards deep layers of the entorhinal cortex (Buzsáki et al., 1992; Buzsáki, 1996). In extracellular field potential recordings, these sharp wave-ripple complexes (SPW-R) appear as monophasic synaptic potentials superimposed by a fast “ripple” oscillation at ~200 Hz (Ylinen et al., 1995).

SPW-R provide a template for sequential activation of selected neurons which repeat previously acquired representations of space- or context-dependent experience (O’Keefe, 1976; Wilson and McNaughton, 1994; Harris et al., 2003). In addition, several studies show that individual cells or units are activated with astonishing temporal precision within individual ripple cycles, which last only ~5 ms (Buzsáki et al., 1992; Ylinen et al., 1995; Csicsvari et al., 1999b). The mechanisms mediating selective and temporally precise activation of hippocampal neurons during such fast oscillations are, however, only partly understood. The intense activation of fast spiking interneurons during SPW-R suggests a role for phasic GABAergic inhibition (Csicsvari et al., 1999b; Klausberger et al., 2003; Ellender et al., 2010; Hájos et al., 2013).

Indeed, repetitive inhibitory postsynaptic potentials can define alternating time windows of enhanced and reduced discharge probability (Traub et al., 2004; Geisler et al., 2005; Mann and Paulsen, 2007). In addition, CA1 pyramidal cells receive ripple-synchronous glutamatergic input (Maier et al., 2011), which may also entrain spikes. This could explain the precise temporal coherence between different hippocampal subfields during propagating SPW-R (Chrobak and Buzsaki, 1996; Bragin et al., 1999; Maier et al., 2003; Both et al., 2008; Memmesheimer, 2010). Lastly, several lines of evidence point towards a role for axo-axonal gap junctions providing a ripple-frequency oscillation within groups of electrically coupled principal cells which would render spike timing relatively independent from the kinetics of IPSPs or EPSPs (Draguhn et al., 1998; Schmitz et al., 2001; Nimmrich et al., 2005; Bähner et al., 2011; Viereckel et al., 2013).

Field potential or EEG recordings provide a spatially weighted average of all intrinsic and synaptic conductance changes detected by the recording electrode. Ion fluxes cause neuronal current sources or sinks which propagate along the dendritic-somatic-axonal axis of the cell and cause balancing currents of opposite sign at locations remote from the site of origin. In principle, lamina-specific recordings of field potentials should therefore be ideally suited to dissect the different components of a complex electrical network event. However, despite the highly ordered laminar structure of hippocampal networks it is still a major challenge to unravel the different components underlying extracellular field potentials (Johnston and Wu, 1995; Csicsvari et al., 2003; Pettersen et al., 2006; Makarova et al., 2011). A major experimental difficulty is given by the critical contribution of many different mechanisms to compound network events which can cause complete disruption of the studied pattern upon systemic application of receptor blockers or other drugs.

Here, we used spatially restricted application of excitatory and inhibitory receptor blockers during multi-laminar recording of SPW-R in CA1 to dissect the differential contribution of GABAergic inhibition and glutamatergic excitation to this highly patterned activity. Using an established *in vitro* model of SPW-R in mouse hippocampal slices we found strong contributions of both, rhythmic inhibition and excitation to ripple oscillations. The power and the leading frequency of rhythmic EPSPs and IPSPs, respectively, differ between different hippocampal layers, reflecting the strongly laminar structure of CA1. While excitatory transmission from upstream CA3 networks seems to be essential for neuronal recruitment, the precise timing depends more critically on inhibition in perisomatic layers. Thus our study reveals complementary functions of simultaneous glutamatergic and GABAergic influences during SPW-R.

## Materials and methods

Experiments were performed on adult male C57Bl6 mice aged 4–8 weeks in compliance with German law and with the approval of the state government of Baden-Württemberg (Nr. T08/10). Brains of CO_2_-anesthesized mice were excised and cooled to 1–4°C in artificial cerebrospinal fluid (ACSF) containing (in mM): 124 NaCl, 3 KCl, 1.8 MgSO_4_, 1.6 CaCl_2_, 10 glucose, 1.25 NaH_2_PO_4_ and 26 mM NaHCO_3_, saturated with 95% O_2_/5% CO_2_ (pH 7.4 at 37°C). After removal of the cerebellum and frontal brain structures, we prepared horizontal slices of 450 μm on a vibratome (VT 1000 S; Leica, Germany). The tissue was allowed to recover for at least 2 h in a Haas-type interface recording chamber at 35 ± 0.5°C before we started the experiments. Most slices used for recordings were from the middle part of the hippocampus.

**Figure 1:**
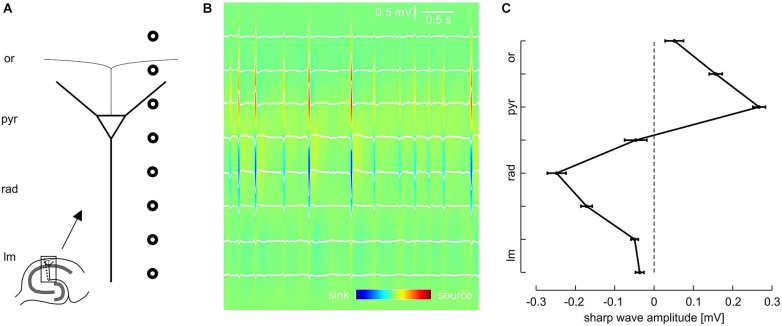
**Laminar profile of SPWs in the CA1 region of mouse hippocampal slices. (A)** Schematic of recording electrode position. **(B)** Representative LFP recordings from eight sites, located as illustrated in **(A)**, and current source density (CSD) plot calculated from the same data. **(C)** Average SPW amplitude across CA1, mean and SEM of 51 slices. Data were pooled according to distance from pyramidal layer. Intervals on y-axis correspond to distance between two recording electrodes.

Field potentials were recorded with bipolar platinum/iridium wires (Science Products, Hofheim, Germany) which were fixed in a line of eight electrodes in a custom-made holder. The distance between individual contacts was approximately 75 μm. This array was positioned perpendicularly to the CA1 pyramidal cell layer (Figure 1A) such that all laminae from alveus to stratum lacunosum-moleculare were covered. Usually, electrodes #5 or #6 were placed over the pyramidal cell layer, as visible from the large positive amplitude of spontaneous sharp waves (see Section Results). Drugs were locally applied by leakage from large glass microelectrodes with tip diameters of ~15 μm. This technique leads to local diffusion of substances into the tissue with a diameter at half maximal concentration of 262 ± 55 μm as has been assessed previously (Bähner et al., 2011). Pipettes were filled with 10 μM gabazine (Tocris Bioscience) or with 200 μM CNQX (Tocris Bioscience; Sachidhanandam et al., 2013; Sato et al., 2014) dissolved in ACSF and were placed on the surface of the slice at about 75 μm distance to the recording electrode in s. radiatum, s. pyramidale or s. oriens. Effects of the respective drugs were assessed 40 min after begin of the local application. For wash-out, the pipette was removed and data was recorded 60 min afterwards.

Field potentials were amplified 100 times by an EXT 10–2F amplifier (npi electronics, Tamm, Germany), low-pass filtered at 2 kHz and digitized at 5–10 kHz (CED1401 interface; Cambridge, UK). Data were sampled with the Spike-2 program (CED) and analyzed with custom-written routines in Matlab (The MathWorks, Natick, MA). Sharp waves were usually detected in s. radiatum from the channel with highest negative event amplitudes. When these events were locally suppressed following drug application to s. radiatum we searched for the largest positive transients in s. pyramidale. Detection threshold was usually 200 μV in low-pass filtered traces (corner frequency 50 Hz). In experiments showing rather low amplitudes threshold was lowered to 100 μV. Slices were excluded if baseline SPW-R frequency was <1 Hz. For statistical analysis, the channel with the largest positive sharp wave amplitude was chosen as representative data for s. pyramidale. Likewise, the channel with the largest negative sharp wave amplitude was chosen as representative data for s. radiatum.

For analysis of high-frequency oscillations (ripples), events underwent continuous wavelet transform (complex Morlet wavelet) starting 33 ms before and ending 67 ms after the peak of a detected sharp wave. We then calculated the peak power of ripples (frequency >140 Hz) and their leading frequency. Current source density (CSD) analysis of field potentials was computed using the spline inverse current source density analysis (iCSD) method (Pettersen et al., 2006). The respective Matlab routine was kindly provided by these authors.

For detection of extracellularly recorded action potentials (“units”), we applied a 500 Hz high-pass filter. Subsequently, single events were extracted by setting a threshold at four times standard deviation (SD) to an “up-only” filtered signal (Cohen and Miles, 2000). This threshold was raised stepwise up to seven times SD if visual inspection of multi-unit activity (MUA) autocorrelation histograms indicated a reduced signal-to-noise ratio. Slices were excluded from MUA analysis when units could not be unambiguously identified. Coupling jitter between units and ripples was calculated based on the width of peaks in the respective cross-correlation as previously described (Both et al., 2008). A similar approach was chosen for analysis of MUA autocorrelation histograms, and coupling jitter was calculated in the same way as for cross-correlograms.

Average data was determined from 5-min sections. In general, quantitative results are given as mean ± SEM or as the first and third quartiles (P25 and P75) if data was not normally distributed. For better visualization, local drug effects are normalized to the baseline value in some figures, whereas statistical significance was computed on the basis of the original values. Parametric tests were used if groups passed a normality test. Otherwise, nonparametric statistics were used. As differential pharmacologic effects were examined, sample size was rather small for each subgroup and data was not normally distributed in many cases. Therefore, nonparametric ANOVA (Friedman test) was conducted throughout this study to compare baseline, wash-in and wash-out condition. *P* values < 0.05 were regarded as significant. If no significant difference was revealed, the *P* value was specified. Otherwise, *post hoc* analysis (Dunn’s multiple comparisons test) was performed and the *P* value of the *post hoc* test was specified.

## Results

Local field potentials (LFPs) were recorded from the CA1 region of 50 mouse hippocampal slices. We regularly observed spontaneous events resembling sharp waves and superimposed fast oscillations (ripples), similar to previous findings from rodents (SPW-R) *in vivo* (Buzsáki et al., 1992; Ylinen et al., 1995) and *in vitro* (Kubota et al., 2003; Maier et al., 2003). In order to dissect the laminar profile of SPW-R we used a linear array of eight equidistant extracellular electrodes which were placed perpendicularly to the pyramidal cell layer of CA1 between s. lacunosum-moleculare and the alveus (Figure 1A).

Previous work indicates that sharp waves in CA1 are generated by synchronous excitatory inputs from CA3 pyramidal cells via the Schaffer collateral (Buzsáki, 1986; Csicsvari et al., 2000; Maier et al., 2003, 2011; Both et al., 2008). In line with this mechanism, the slow component of the spontaneous local field potential transients revealed a strong negative deflection in s. radiatum (Figure 1B). In contrast, sharp waves were positive-going in s. pyramidale. Analysis of all eight recording positions confirmed this phase reversal between dendritic and somatic layers, with very low sharp wave amplitudes in the extreme positions (s. lacunosum-moleculare and s. oriens, respectively; Figure 1C). Current source density analysis (Mitzdorf, 1985; Pettersen et al., 2006) revealed pronounced current sinks in s. radiatum as well as current sources in s. pyramidale. This data is consistent with the reported excitatory input to the proximal dendritic layer and simultaneous perisomatic inhibition (Ylinen et al., 1995; Ellender et al., 2010; Maier et al., 2011). This hypothesis was subsequently tested by lamina-specific application of glutamatergic and GABAergic receptor antagonists, respectively.

**Figure 2:**
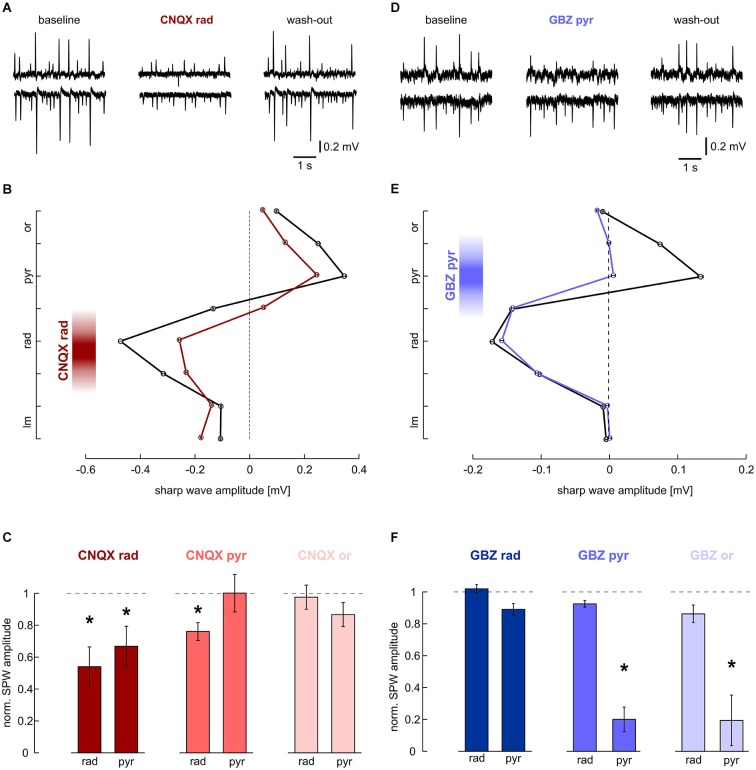
**Effects of local application of 200 μM CNQX and 10 μM gabazine on SPWs. (A, B)** Local application of CNQX to s. radiatum reversibly inhibits SPW in CA1. Note that in this slice, SPW amplitude was reduced in both layers. **(A)** LFP recordings, representative of s. pyramidale (upper trace) and s. radiatum (lower). (Left) baseline, (middle) after 40 min of local wash-in, (right) after 60 min wash-out. **(B)** Average SPW amplitude across CA1 before (black) and after (dark red) local application of CNQX to s. radiatum. Mean and SEM of *n* = 646 (baseline) and *n* = 253 (wash-in) sharp waves from one slice. Intervals on y-axis correspond to distance between two recording electrodes. **(C)** Normalized SPW amplitude in s. radiatum and s. pyramidale after local application of CNQX to (left) s. radiatum (*n* = 7 slices; s. radiatum: *P* < 0.01, s. pyramidale: *P* < 0.05), (middle) s. pyramidale (*n* = 6 slices; s. radiatum: *P* < 0.05, s. pyramidale: *P* > 0.05) and (right) s. oriens (*n* = 7 slices; s. radiatum: *P* > 0.1, s. pyramidale *P* > 0.1). **(D, E)** Local application of gabazine to s. pyramidale reversibly reduces SPW amplitude specifically in this layer. Note that in s. radiatum event amplitude and frequency are not affected. **(D)** LFP recordings, representative of s. pyramidale (upper trace) and s. radiatum (lower). (Left) baseline, (middle) after 40 min of local wash-in, (right) after 60 min wash-out. **(E)** Average SPW amplitude across CA1 before (black) and after (blue) local application of gabazine to s. pyramidale. Mean and SEM of *n* = 757 (baseline) and *n* = 494 (wash-in) sharp waves from one slice. Intervals on y-axis correspond to distance between two recording electrodes. **(F)** Normalized SPW amplitude in s. radiatum and s. pyramidale after local application of gabazine to (left) s. radiatum (*n* = 11 slices; s. radiatum: *P* > 0.05, paired *t* test, s. pyramidale: *P* > 0.05, Dunn’s multiple comparisons test), (middle) s. pyramidale (*n* = 6 slices; s. radiatum: *P* > 0.1, s. pyramidale: *P* < 0.01) and (right) s. oriens (*n* = 8 slices; s. radiatum: *P* > 0.1, s. pyramidale: *P* < 0.01).

A major fraction of excitatory synaptic inputs was antagonized with the AMPA/kainate glutamate receptor antagonist CNQX (200 μM). When applied to s. radiatum, CNQX reversibly reduced sharp wave amplitude in s. radiatum (Figures 2A,B). At the same time, SPW-R frequency in s. radiatum decreased (1.49 ± 0.16 Hz at baseline, 0.68 ± 0.21 Hz after local wash-in and 0.91 ± 0.18 Hz after wash-out; *n* = 7 slices; *P* < 0.01). In one out of seven slices SPW-R was completely abolished and started to recover after ~2 min of drug washout. Field potential amplitudes in s. pyramidale were also significantly reduced. Conversely, when we applied CNQX to the pyramidal cell layer, sharp wave amplitude was stable in this layer, but showed a slight reduction in s. radiatum. In contrast to these findings no significant change of sharp wave amplitude or frequency was noted following application of CNQX in s. oriens (Figure 2C). Together, these results indicate that sharp waves are indeed generated by a lamina-specific excitatory input to s. radiatum.

The positive-going sharp waves in s. pyramidale can be generated in at least two different ways: they may reflect balance currents following excitatory input to the dendrites or, alternatively, arise from outward currents generated by inhibition within the pyramidal cell layer itself (Johnston and Wu, 1995 pp. 426–434; Ylinen et al., 1995). We therefore applied the GABA_A_ receptor antagonist gabazine (10 μM) to s. pyramidale. As a result, the positive field potential deflection in s. pyramidale was strongly diminished while the negative-going transient in s. radiatum remained unaffected (Figures 2D,E). In two of six slices, the transient in s. pyramidale reversed and we recorded negative deflections after local wash-in of the drug. When the GABAergic antagonist was applied to s. oriens, effects were very similar to those observed after disinhibiting s. pyramidale. Application of gabazine in s. radiatum had no significant effects (Figure 2F). These results indicate the lamina-specific contribution of GABAergic inhibition in s. pyramidale and oriens to sharp waves.

**Figure 3:**
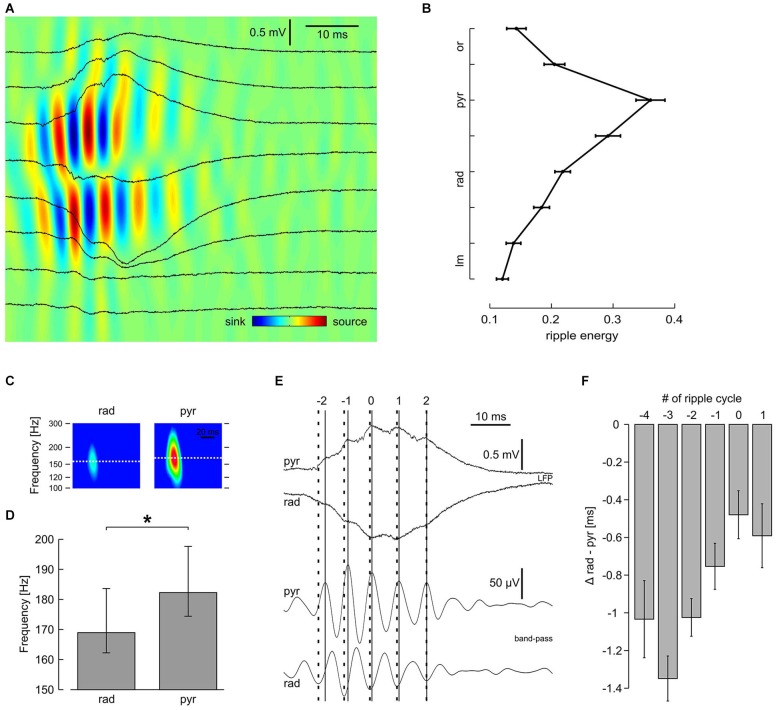
**Laminar profile of high-frequency oscillation reveals distinct properties in s. pyramidale and s. radiatum. (A)** Original traces and CSD plot showing one representative SPW-R. **(B)** Average ripple energy across CA1; mean and SEM of 51 slices. Data were pooled according to distance from pyramidal layer. Intervals on y-axis correspond to distance between two recording electrodes. **(C)** Representative spectrograms of *n* = 540 SPW-R from one slice. **(D)** Average ripple frequency in s. radiatum and s. pyramidale; median, 25th and 75th quantiles of *n* = 38 slices; *P* < 0.01, Wilcoxon matched-pairs signed-ranks test. **(E)** (Upper) original and (lower) band-pass filtered traces, representative of s. pyramidale and s. radiatum, showing one SPW-R. Note that the interval between ripple troughs in s. pyramidale (solid lines) and corresponding peaks in s. radiatum (bold dashed lines) decreases during the course of this SPW-R. Index 0 is ascribed to the ripple trough left of the sharp wave peak detected in s. pyramidale. **(F)** Average phase lag of high-frequency oscillation in s. radiatum in relation to s. pyramidale, mean and SEM of *n* = 51 slices. Regression line for cycle #−4 to #1: average slope 0.16 ms per cycle, r^2^ 0.074, *P* < 0.01; cycle #−3 to #0: average slope 0.29 ms per cycle, r^2^ 0.13, *P* < 0.01.

Sharp waves in CA1 were regularly superimposed by fast oscillations, reminiscent of hippocampal ripples *in vivo* (Buzsáki et al., 1992) and *in vitro* (Maier et al., 2003). These network oscillations were most pronounced in s. pyramidale but could also clearly be identified in the apical dendritic layer (s. radiatum) and in the proximal part of the basal dendritic layer (s. oriens; Figure 3A). The laminar distribution of ripple energy revealed a continuous decay between s. pyramidale and s. lacunosum-moleculare and a similar, though much steeper decay in s. oriens (Figure 3B). Current source density analysis confirmed the rapid interplay between sinks and sources in s. pyramidale and also in s. radiatum (Ylinen et al., 1995; Sullivan et al., 2011). Interestingly, the ripples had slightly lower frequency in s. radiatum as compared to s. pyramidale (Figure 3C). We therefore examined—with respect to the ripple oscillation in the pyramidal layer—the phase of fast oscillations recorded in s. radiatum during the course of SPW-R (Figure 3D). Interestingly, ripple troughs in s. radiatum preceded corresponding peaks in s. pyramidale significantly at the beginning of a SPW-R. Towards the end of a SPW-R, this phase shift decreased systematically (Figures 3E,F). Thus, frequencies of ripples are not uniform across CA1, allowing complex temporal interactions between dendritic and somatic layers.

**Figure 4:**
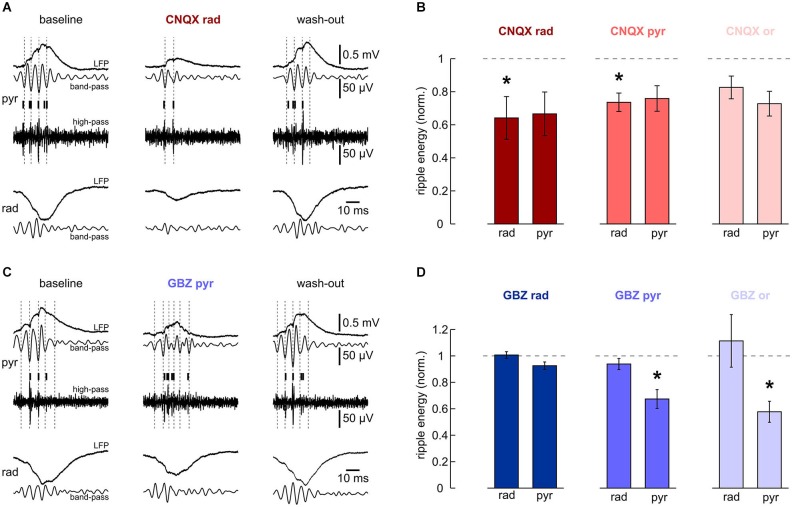
**Effects of local application of 200 μM CNQX and 10 μM gabazine on high-frequency oscillations**. **(A, B)** Local application of CNQX to s. radiatum reversibly inhibits fast oscillations in this layer. Note that in this slice, ripples in s. pyramidale were also affected. **(A)** Representative LFP recordings, band-pass and high-pass filtered, from s. pyramidale (upper traces) and s. radiatum (lower). Detected units are indicated by vertical bars. (Left) baseline, (middle) after 40 min of local wash-in, (right) after 60 min wash-out. **(B)** Normalized ripple energy in s. radiatum and s. pyramidale after local application of CNQX to (left) s. radiatum (*n* = 7 slices; s. radiatum: *P* < 0.05, s. pyramidale: *P* > 0.05), (middle) s. pyramidale (*n* = 6 slices; s. radiatum: *P* < 0.05, s. pyramidale: *P* > 0.05, Dunn’s multiple comparisons test) and (right) s. oriens (*n* = 7 slices; s. radiatum: *P* > 0.05, Dunn’s multiple comparisons test, s. pyramidale: *P* > 0.05, Dunn’s multiple comparisons test). **(C, D)** Local application of gabazine to s. pyramidale or s. oriens reversibly inhibits ripples in the pyramidal layer. Note that ripple energy in s. radiatum is not affected. **(C)** Representative LFP recordings, band-pass and high-pass filtered, from s. pyramidale (upper traces) and s. radiatum (lower). Detected units are indicated by vertical bars. (Left) baseline, (middle) after 40 min of local wash-in, (right) after 60 min wash-out. **(D)** Normalized ripple energy in s. radiatum and s. pyramidale after local application of gabazine to (left) s. radiatum (*n* = 11 slices; s. radiatum: *P* > 0.1, s. pyramidale: *P* > 0.05), (middle) s. pyramidale (*n* = 6 slices; s. radiatum: *P* > 0.1, s. pyramidale: *P* < 0.01), and (right) s. oriens (*n* = 8 slices; s. radiatum: *P* > 0.1, Wilcoxon matched-pairs signed-ranks test, s. pyramidale: *P* < 0.01).

We next analyzed effects of CNQX and gabazine on ripple oscillations. Based on the laminar differences in ripple phase and frequency described above, we looked for different contributions of synaptic excitation and inhibition in the respective layers. Glutamatergic transmission was suppressed by local application of CNQX to s. radiatum, pyramidale or oriens, respectively. Despite a tendency to reduced ripple energy in all layers (Figure 4B), significant effects were layer-specific. Application of CNQX to s. radiatum clearly suppressed the fast oscillation within the same layer while an apparent reduction in s. pyramidale was not significant (Figures 4A,B). Application of CNQX to the pyramidal layer also attenuated ripples in s. radiatum, though this effect was less pronounced. No significant effects were observed following application in s. oriens (Figure 4B). These results indicate that glutamatergic transmission in s. radiatum contributes significantly to high-frequency oscillations in this layer.

In addition, we examined the role of synaptic inhibition for ripples. Application of gabazine to s. pyramidale or s. oriens consistently reduced ripple energy in s. pyramidale (Figures 4C,D). Following application to s. radiatum, however, no significant effects on ripples were observed. Interestingly, high-frequency oscillations within s. radiatum were unaffected by gabazine, even upon application within the same layer (Figure 4D). Together, these data support the importance of rhythmic perisomatic or proximal-dendritic GABAergic inhibition for SPW-R (Ylinen et al., 1995).

Laminar block of excitatory and inhibitory transmission had differential effects on ripple frequency. Application of CNQX to s. radiatum had no significant impact on its median (181 ± 10 Hz at baseline, 178 ± 11 Hz after local wash-in and 185 ± 12 Hz after wash-out; *n* = 6 slices, *P* > 0.1) and variability. In contrast, ripple frequency variability was strongly increased following application of gabazine to s. pyramidale (semi-quartile range: 30.3 ± 4.6 Hz at baseline, 114.3 ± 20.4 Hz after local wash-in and 39.9 ± 5.7 Hz after wash-out; *n* = 6 slices, *P* < 0.05). Its median showed a tendency to increase (179 ± 9 Hz at baseline, 209 ± 22 Hz after local wash-in and 180 ± 10 Hz after wash-out; *n* = 6 slices, *P* > 0.05). These findings underline the key role of phasic inhibition for synchronization of fast oscillations during SPW-R (Buzsáki et al., 1992; Ylinen et al., 1995) specifically in s. pyramidale and s. oriens (Bähner et al., 2011).

**Figure 5:**
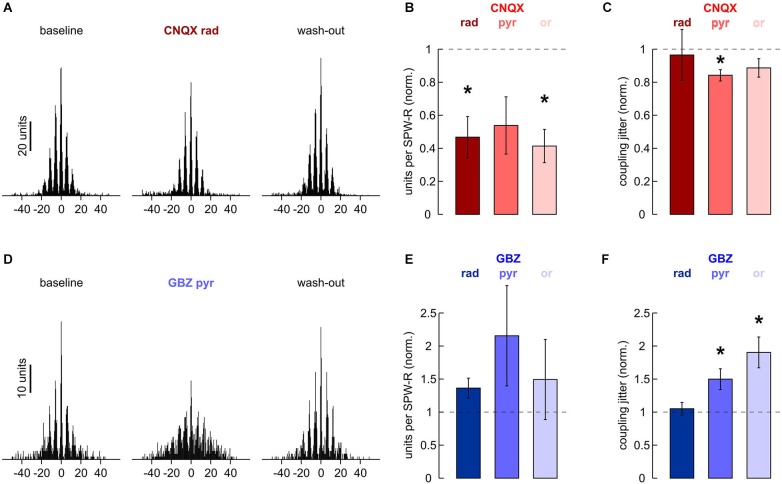
**Effects of local application of 200 μM CNQX and 10 μM gabazine on MUA. (A)** Cross-correlation histogram of *n* = 2170 units and *n* = 796 ripple troughs in s. pyramidale. (Left) baseline, (middle) after 40 min of local wash-in, (right) after 60 min wash-out. **(B)** Normalized MUA frequency in s. pyramidale during SPW-R after local application of CNQX to (left) s. radiatum (*n* = 7 slices; *P* < 0.01), (middle) s. pyramidale (*n* = 6 slices; *P* > 0.05) and (right) s. oriens (*n* = 7 slices; *P* < 0.01). Note that MUA frequency during SPW-R was reduced in five out of six slices after application to s. pyramidale. **(C)** Normalized coupling jitter of MUA to ripples in s. pyramidale after local application of CNQX to (left) s. radiatum (*n* = 5 slices; *P* > 0.1), (middle) s. pyramidale (*n* = 5 slices; *P* < 0.05) and (right) s. oriens (*n* = 7 slices; *P* > 0.1). **(D)** Cross-correlation histogram of *n* = 291 units and *n* = 680 ripple troughs in s. pyramidale. (Left) baseline, (middle) after 40 min of local wash-in, (right) after 60 min wash-out. **(E)** Normalized MUA frequency in s. pyramidale during SPW-R after local application of gabazine to (left) s. radiatum (*n* = 7 slices; *P* > 0.1), (middle) s. pyramidale (*n* = 6 slices; *P* > 0.05) and (right) s. oriens (*n* = 6 slices; *P* > 0.1). **(F)** Normalized coupling jitter of MUA to ripples in s. pyramidale after local application of gabazine to (left) s. radiatum (*n* = 7 slices; *P* > 0.1), (middle) s. pyramidale (*n* = 6 slices; *P* < 0.05) and (right) s. oriens (*n* = 6 slices; *P* < 0.01).

Network oscillations have been suggested to entrain action potentials of multiple neurons into a common rhythm (Buzsáki and Draguhn, 2004). During SPW-R, in particular, unit discharges in s. pyramidale are tightly coupled to ripple troughs, as can be seen in cross-correlation histograms (Buzsáki et al., 1992; Figure 5A). Autocorrelation histograms underline this observation. They show peaks at intervals of about 5 ms, confirming periodic changes in discharge probability at ripple frequency (Csicsvari et al., 1999b). We tried to dissect the impact of different synaptic components on MUA.

Apparently, application of CNQX to any of the three layers tested interfered with recruitment of units in s. pyramidale. This effect was significant after application to s. radiatum and s. oriens (Figure 5B). Coupling jitter, which was calculated from cross-correlation between MUA and field ripples, remained stable or was slightly reduced (Figure 5C). MUA autocorrelation histograms also remained largely unaffected. After application of CNQX to s. radiatum, coupling jitter was 40.8 ± 3.7% at baseline, 38.8 ± 4.5% after local wash-in and 44.7 ± 4.5% after wash-out (*n* = 4 slices). Similar effects were observed after CNQX had been applied to s. pyramidale or s. oriens.

Finally, we examined if local application of gabazine would manipulate unit discharge behavior. In s. pyramidale, MUA frequency was not significantly affected (Figure 5E). Coupling of MUA to ripple troughs, however, was reversibly impaired (Figures 5D,F). Analysis of autocorrelation histograms further indicated that unit firing got more disperse. Coupling jitters were 28.9 ± 0.8% at baseline, 40.2 ± 2.6% after local wash-in and 25.1 ± 1.7% after wash-out (*n* = 4 slices). Interestingly, application to s. oriens yielded very similar effects. Autocorrelation coupling jitter was 31.0 ± 2.0% at baseline, 43.7 ± 2.3% after local wash-in and 34.8 ± 3.3% after wash-out (*n* = 5 slices, *P* < 0.05). In contrast, no significant changes in MUA frequency, cross- or autocorrelation were observed after gabazine had been applied to s. radiatum. The precision of action potential timing thus crucially depends on inhibitory currents in s. pyramidale and s. oriens (Buzsáki et al., 1992; Ylinen et al., 1995) specifically in s. pyramidale and s. oriens.

## Discussion

Sharp wave-ripple complexes (SPW-R) reflect highly ordered activity patterns, which are believed to support specific cognitive functions like memory consolidation (Buzsáki, 1989). The underlying cellular mechanisms have been studied both *in vivo* (Buzsáki et al., 1992) and the *in vitro* slice preparation (Maier et al., 2003). These studies have shown that perisomatic inhibition is of key importance (Ylinen et al., 1995; Ellender et al., 2010) and that, at the same time, glutamatergic inputs from upstream projection neurons mediate synaptic excitation and propagation of activity (Buzsáki, 1986; Csicsvari et al., 2000; Both et al., 2008; Maier et al., 2011).

SPW-R are recorded as an LFP in s. pyramidale during behavioral states of awake immobility, originally called large irregular activity (Vanderwolf, 1969). These transient field potentials have typical waveforms, laminar profiles and propagation patterns (Buzsáki et al., 1992; Ylinen et al., 1995; Chrobak and Buzsaki, 1996) which are also visible in hippocampal slice preparations (Kubota et al., 2003; Behrens et al., 2005; Both et al., 2008; Ellender et al., 2010). The relationship, however, between such extracellular compound potentials and the underlying excitatory and inhibitory synaptic currents, action potentials and other processes in multiple cells, is not trivial. In fact, LFPs result from a large variety of local and remote currents, including balance currents between different layers and far-reaching effects from remote current sinks and sources (Herreras, 1990; Johnston and Wu, 1995 pp. 426–434; Sirota et al., 2008; Kajikawa and Schroeder, 2011). The trilaminar anatomy of the cornu ammonis, which is formed by a line of multiple equally oriented cells (“open field” arrangement; Johnston and Wu, 1995 pp. 428 f.), provides ideal conditions to untangle the lamina-specific mechanisms underlying field potential deflections. Here we made use of a hippocampal slice preparation that preserves network activity patterns while allowing for flexible pharmacologic manipulation without systemic side-effects. Interpretation of our results should take into account that field potential recordings can be affected by potential fluctuations in remote areas. Diffusion of the drugs following local application is, however, more restricted. Therefore, pharmacological effects may have been underestimated as compared with bath application of drugs. Nevertheless, we demonstrate that glutamatergic and GABAergic receptor antagonists exert different and lamina-specific effects on sharp waves and superimposed ripple oscillations in CA1. Our data confirm a major excitatory input in s. radiatum which provides synaptic excitation at a different (lower) frequency than the resulting local network ripple within CA1. Moreover, both sharp waves and superimposed ripples are generated by both, excitatory and inhibitory inputs, with different contributions of either mechanism in different laminae.

We report that local application of CNQX suppressed sharp waves in s. radiatum. This finding indicates that the corresponding sink is largely generated by synchronous activation of AMPA/kainate receptors from Schaffer collateral afferents (Buzsáki, 1986; Csicsvari et al., 2000; Both et al., 2008). A critical involvement of NMDA receptors seems unlikely, as SPW-R are insensitive to 2-amino-5-phosphonopentanoic acid (APV) under our recording conditions (unpublished finding). We cannot exclude that the high concentration of CNQX close to the tip of the application pipette did also affect GABA_A_ receptor-mediated currents, as previously reported (McBain et al., 1992; Maccaferri and Dingledine, 2002). Local GABAergic potentials in s. radiatum, however, would be expected to generate positive field potential transients within the same layer, in contrast to our finding of reduced negative transients. Conversely, SPW-R amplitude in s. pyramidale was reduced following local application of gabazine. In some slices we recorded negative transients in the pyramidal layer. The source in the pyramidal layer thus seems to be largely due to active GABAergic outward currents (Ylinen et al., 1995), presumably evoked by parvalbumin-positive basket cells. These interneurons target the perisomatic compartment of CA1 pyramidal cells and are highly active during SPW-R (Ylinen et al., 1995; Csicsvari et al., 1999b; Klausberger et al., 2003; Bähner et al., 2011). Our data could, however, not demonstrate the contribution of passive return currents, as had been discussed previously (Ylinen et al., 1995). Thus, sharp waves in different layers are generated by clearly different processes which can be pharmacologically distinguished: synaptic excitation in the dendritic cell layer and synaptic inhibition in perisomatic regions. As a complicating factor, application of CNQX to s. radiatum suppressed sharp waves in s. pyramidale as well. This effect could be ascribed to a reduction of feed-forward inhibition (Gulyás et al., 1999; Pouille and Scanziani, 2001). In summary, our results indicate that the laminar profile of SPW-R is predominately evoked by local active sinks and sources, respectively, rather than remote passive ones (Herreras, 1990; Johnston and Wu, 1995 pp. 426–434).

How is the superimposed high-frequency oscillation generated? Previous work based on recordings from individual neurons *in vivo* (Ylinen et al., 1995; Csicsvari et al., 1999b) and *in vitro* (Bähner et al., 2011; Maier et al., 2011) suggests a key role for GABAergic interneurons, again presumably parvalbumin-positive basket cells. Those cells target the perisomatic compartment of CA1 pyramidal cells, show fast spiking strongly coupled to field ripples (Klausberger et al., 2003) and have therefore been proposed to generate the current sources at ripple frequency observed in s. pyramidale (Ylinen et al., 1995). Indeed, we observed that local application of gabazine reduces ripple energy in s. pyramidale. This suggests a substantial contribution of the predicted synchronous GABAergic currents. Effects on the sharp wave component seemed more pronounced, which could indicate that local excitatory currents in s. radiatum (Maier et al., 2011) also have an impact on ripples in the pyramidal layer. It should be noted, however, that ripple energy is calculated as an integral of the continuous wavelet transform. Therefore, it might be considerably greater than zero even at baseline level, apparently attenuating drug effects. Moreover, high-frequency oscillations in the wavelet transform may contain rhythmically entrained unit activity which is still present after application of gabazine. In addition, our data indicate that GABAergic currents in s. oriens, which might tune axonal excitability, contribute to ripple oscillations. This would be consistent with an involvement of hypothesized axo-axonic gap junctions that allow ectopic action potential genesis (Bähner et al., 2011; Traub et al., 2012). Experimental work and modeling studies also indicate that phasic inhibition is crucial for the tight phase locking of pyramidal cell action potentials to ripples (Buzsáki et al., 1992; Bähner et al., 2011). Indeed, block of GABA_A_ receptors strongly interfered with the coupling of pyramidal layer MUA to field ripples. This confirms that during SPW-R, fast-spiking interneurons act as a clock that precisely tunes the timing of principal cell discharges. Synchronous action potentials, in turn, might also contribute to the shape of high-frequency oscillations recorded in the pyramidal layer (“mini” population spikes Buzsáki, 1986; Ylinen et al., 1995).

Ripples have thus been a phenomenon primarily linked to the pyramidal layer. Initially, alternating sinks and sources were depicted as being confined to it (Ylinen et al., 1995). Recently, however, the coexistence of a concomitant high-frequency oscillation in s. radiatum has been reported *in vivo* (Sullivan et al., 2011). *In vitro* we observed a similar laminar CSD profile, depicting a fast oscillation of relevant energy also in s. radiatum. Interestingly, this oscillation is characterized by a slightly, but distinctively lower frequency. It is thus unlikely a mere epiphenomenon of ripples in the pyramidal layer. These radiatum “ripples” rather seem to be evoked by precisely timed local glutamatergic currents, as evidenced by their sensitivity to CNQX. Those might be elicited by Schaffer collateral inputs, considering that CA3 initiates SPW-R (Buzsáki, 1986) and shows slower high-frequency oscillations (Csicsvari et al., 1999a; Maier et al., 2003; Both et al., 2008; Sullivan et al., 2011). Though a recent study—without subregional coherence analysis—concludes that ripples are not transferred wave by wave (Sullivan et al., 2011), the tight cross-correlation between single CA3 pyramidal cells and ripples in CA1 has been well-documented, especially for corresponding subregions (Csicsvari et al., 2000; Both et al., 2008). The CA3 network thus generates a highly-synchronized output pattern rather than providing diffuse excitation onto CA1 pyramidal cells. This signal very likely contains some frequency component slightly below the typical ripple spectrum, and should substantially contribute to high-frequency oscillations in CA1 s. radiatum. Nevertheless, this downstream CA1 network has intrinsic properties that allow the generation of ripples, as evidenced by recordings from CA1 minislices (Maier et al., 2003). The interplay between excitatory and inhibitory events has recently been directly demonstrated by whole-cell recordings from CA1 pyramidal cells. These show that excitation is phase-advanced at the beginning of SPW-R and a progressive synchronization with inhibition towards the end of each complex (Maier et al., 2011). Interestingly, we observed an analogous phase shift between ripple troughs (s. radiatum) and corresponding peaks (s. pyramidale), which progressively decreased during the course of individual SPW-R. This finding is consistent with a slower frequency in s. radiatum and underlines the existence of an additional high-frequency oscillation distinct from ripples in the pyramidal layer. In conclusion, CA3 principal neurons could assist in suprathreshold excitation of downstream neurons forming a cell assembly (Harris et al., 2003) via precisely timed and spatially confined currents. On the other hand, the decreasing phase-shift between excitation and inhibition tightens the temporal window for spike generation towards the end of an indiviual SPW-R and hence might contribute to the termination of this sharply delineated network burst.

Though the relationship between LFP waveforms and the underlying multi-neuronal activity patterns may be complex (Henze et al., 2000; Csicsvari et al., 2003; Pettersen et al., 2006)—during SPW-R, they reflect a characteristic signature of different neuronal assemblies (Reichinnek et al., 2010). Our data indicate that SPW-R recorded in CA1 mainly reflect a weighted average of well-coordinated local synaptic currents. At least two distinct sources of high-frequency oscillations can be distinguished: in s. pyramidale, they seem due to GABAergic inputs from local interneurons, while in s. radiatum, the specific waveform is largely evoked by long-range glutamatergic afferents, likely from CA3. In addition, experimental and theoretical approaches suggest that supralinear dendritic interactions (Memmesheimer, 2010) and ectopic action potential generation (Bähner et al., 2011; Traub et al., 2012) might play a role in assembly formation.

## Conflict of interest statement

The authors declare that the research was conducted in the absence of any commercial or financial relationships that could be construed as a potential conflict of interest.
